# Linear or circular: Anastomotic ulcer after gastric bypass surgery

**DOI:** 10.1007/s00464-021-08597-6

**Published:** 2021-06-18

**Authors:** Aline Schäfer, Philipp Gehwolf, Katrin Kienzl-Wagner, Fergül Cakar-Beck, Heinz Wykypiel

**Affiliations:** Department of Visceral-, Transplant- and Thoracic Surgery, Medical University of Innsbruck, Anichstrasse35, 6020 Innsbruck, Austria

**Keywords:** Anastomotic ulcer, Marginal ulcer, Laparoscopic gastric bypass, Metabolic surgery, Bariatric surgery

## Abstract

**Background:**

After laparoscopic Gastric Bypass Procedure (GBP), anastomotic ulcers (AU) at the gastrojejunostomy (GJ) occur in up to 16% of the patients. Surgical techniques seem to influence the development of AU, but this is still a matter of discussion. This study aims to compare the incidence of AU in circular-stapled (CS) versus linear-stapled (LS) gastrojejunostomy.

**Methods:**

Single-centre retrospective analysis of 241 (m 77 /f 164) consecutive patients (126 CS, 115 LS) with primary or revisional GBP including Roux-Y-Gastric Bypass (RYGB) and One-Anastomosis Gastric Bypass (OAGB) between 01/2014 and 01/2018. Follow-up with oesophagogastroduodenoscopy was only performed in symptomatic patients. Age, body mass index (BMI), comorbidities, smoking and medication were analyzed in both groups. The data are reported as total numbers (%) and mean ± standard deviation.

**Results:**

AU occurred significantly more often in the CS group than in the LS group (*p* = 0.0034). Moreover, refractory AU and the need for revisional surgery were higher in the CS group. Smoking correlates significantly with the development of AU, whereas other risk factors had no impact on its incidence.

**Conclusion:**

Linear-stapled gastrojejunostomy with a long and narrow pouch should be the preferable procedure for reducing AU development risk. Smoking cessation minimizes the risk for AU and is a necessary part of the treatment.

Laparoscopic Gastric Bypass (GBP) is a safe and effective treatment for obesity and its comorbidities [1, 2]. It is one of the most common bariatric surgeries worldwide and thus, permanent improvement of the surgical technique is necessary to reduce the number of intra- and postoperative complications. In long-term studies, we still observe postoperative complications that require revisional surgery, such as internal herniation, chronic reflux, malnutrition, anastomotic leakage, strictures and anastomotic ulcerations (AU). The latter is reported in up to 16%, but the incidence may be even higher when AU are subclinical and remain undetected [3, 4]. AU can cause chronic epigastric pain, reflux or digestive disorders and is primarily treated conservatively. When conservative treatment fails, a revision of the gastrojejunostomy (GJ) is necessary and patients sometimes even require emergency surgery when perforation occurs [3, 5, 6].

To this point, surgical technique (circular-stapled vs. linear-stapled vs handsewn, size and configuration of the pouch, usage of non-absorbable sutures), chronic gastric disease (helicobacter pylori infection, gastritis), comorbidities (smoking, type 2 diabetes (T2D), hypertension (HTN)), demographic factors (weight, gender and age) and the prophylactic use of proton pump inhibitors (PPI) are still a matter of discussion for the development of AU [7–13].

To date, a general statement of AU treatment does not exist. Good results have been shown in treatment with PPIs. However, there are no clear recommendations of dose (prophylactic vs therapeutic), duration, single agent or the combination with sucralfate or H2 blockers. Smoking cessation and the control of comorbidities, such as T2D and HTN should be part of the therapy [14]. Still, it is also not clear which patients profit from revisional surgery [7, 15].

## Objectives

In this retrospective study, we compare the impact of circular- and linear-stapled gastrojejunostomy on AU development incidence. This study's secondary endpoints are demographic factors, comorbidities (smoking, T2D, HTN, use of nonsteroidal anti-inflammatory drugs (NSAIDs)) and preoperative findings in oesophagogastroduodenoscopy (EGD) as risk factors of developing an AU.

### Patients and methods

In this retrospective single-centre study, all 241 consecutive patients who underwent laparoscopic GBP (either One-Anastomosis Gastric Bypass (OAGB) or Roux-en-Y-Gastric Bypass (RYGB)), including revisional surgeries at a tertiary hospital between 01/2014 and 12/2017 were analyzed.

Each group's data included demographic and medical aspects, such as age, sex, Body Mass Index (kg/m^*2*^, BMI), smoking behavior, daily medication, comorbidities, surgical technique and peri- and postoperative complications. The data are depicted as total numbers (%), mean and standard deviation. *p* values were calculated with the Chi-square test or Fisher's exact test. A *p* value < 0.05 was considered to be significant.

From 01/2014 to 01/2016, the gastrojejunostomy was performed using a circular stapling (CS) technique and from 01/2016 to 12/2017, a linear-stapled (LS) gastrojejunostomy was performed. All bypasses were constructed in an antecolic and antegastric fashion. Our department switched from CS to LS for creating the gastroenterostomy mainly due to better handling of the linear stapler and, beyond that, avoiding a longer skin incision prone to infection, shorter operation times, skipping the involvement of anaesthesiologists in anvil placement, as well as fewer costs seemed attractive. There are also recommendations from two meta-analyses [16, 17]. Simultaneously, the stapler system's change came along with a change of pouch size and limb length. Moreover, with establishing the OAGB at our department, the complete operation technique in all GBP has been unified to LS gastrojejunostomy as described by Prager [18].

In this study, we also included patients with revisional surgery after laparoscopic Adjustable Gastric Banding (LAGB): They received a revisional GBP (RYGB or OAGB) either in a one-stage procedure with simultaneous band removal or a two-stage procedure with GBP after previous band removal [19].

Selection criteria for laparoscopic One-Anastomosis Gastric Bypass (OAGB) were more than 45 years of age and no evidence of gastroesophageal reflux. OAGB may be associated with biliary reflux and in some cases, oesophagogastric cancer may develop [20–22]. Thus, physiological findings in EGD and manometry, indicating a competent lower oesophageal sphincter, were mandatory for undergoing OAGB surgery. The limit of 45 years was chosen to minimize exposition time for presumed gastric cancer development due to biliary acid exposure [22]. The length of the loop in OAGB was individually adapted on total small bowel length and BMI. In patients with BMI > 50 kg/m^2^, the loop was 200 cm, but shorter in patients with a BMI < 50 kg/m^2^. The gastrojejunostomy in OAGB was always created with a linear stapler [18].

### Preoperative workup

Preoperative workup was performed according to the International Federation for Surgery of Obesity and Metabolic Disorders (IFSO) [23] recommendations, including a complete metabolic workup, a nutrition consultation [24], a psychological consultation as well as an EGD with biopsies. In the case of a Helicobacter pylori (H. pylori) infection, H. pylori eradication was performed preoperatively. Preoperative workup also included oesophageal manometry either with water-perfused stationary pull-back technique or high-resolution technique and 24-h pH impedance monitoring [25]. In those who could not tolerate the manometry probe, a videofluoroscopic swallowing examination was performed instead. All operations were performed by a specialized team of bariatric surgeons under general anaesthesia in Lithotomy (French) and Anti-Trendelenburg position, with a four- or five-trocar technique. First, the small bowel's total length was counted for measuring the length of the alimentary and biliary limb: When the small bowel's length was more than 350 cm, both OAGB and RYGB were feasible. In revisional surgery cases after LAGB, the anatomic changes due to the band were meticulously reversed to obtain quite normal anatomy of the stomach, including removing the band channel. In the next step, either a circular-stapled or a linear-stapled anastomosis was created.

### Surgical technique

In patients with circular-stapled anastomosis, a short (5–8 cm) and wide (3–4 cm) pouch was created. The anvil was inserted transorally using the OrVil ™ system and a circular-stapled end-to-side gastrojejunostomy of 25 mm (CEEA, Covidien Inc., Intl.). The jejuno-jejunostomy was created with a 40–45 mm longitudinal side to side stapled anastomosis with a biliary limb of 100 cm and an alimentary limb of 150 cm. The insertion sites were closed by absorbable running sutures. The surgical technique was described in detail elsewhere [26].

In those patients with linear-stapled anastomosis, the lesser sac was opened close to the lesser curvature at the crow’s foot position. An endoscopic stapler loaded with a 45-mm/3.5-mm cartridge (Endo-GIA®, Covidien, USA or Echelon®, Ethicon Inc., Intl.) was used for sectioning the stomach horizontally and thereafter creating the pouch. This was calibrated with a 35 French (1.2 cm) gastric tube. A linear-stapled gastrojejunostomy of 35–40 mm with a biliary length of 150 cm and an alimentary length of 50 cm was created. The insertion sites were closed by absorbable running barbed sutures. The jejuno-jejunostomy was created with a 40–45 mm longitudinal stapler and the insertion site was closed with a handsewn running barbed suture.

The jejuno-jejunal mesenteric gap and the Peterson's space were consistently closed with non-absorbable running barbed sutures. Air impermeability of gastric anastomosis was proven by intraoperative gastroscopy with an underwater air leak test. If necessary, additional sutures were placed. Simultaneously, the pouch length from the end of the gastric folds to the anastomosis was routinely measured (approx. 9–11 cm) in gastroscopy.6/10/2021 11:26:00 AM.

### Postoperative follow-up

Upper-GI series with water-soluble contrast media was performed routinely on postoperative day two. All patients received a nutritional consultation before discharge. Patients were scheduled for a metabolic consultation three months, six months and then annually. A routine surgical follow-up examination without routine EGD was scheduled at three and twelve months postoperatively or whenever symptoms occurred. Indications for an EGD on follow-up were reflux symptoms, epigastric pain, dyspepsia, digestive disorders or melena. In the case of an AU, conservative treatment was initiated, including a therapeutic dose of PPI, sucralfate, smoking cessation, H. pylori eradication when tested positive and an EGD follow-up six to eight weeks after. If the AU was still existent and showed no healing tendency on follow-up, the PPI was converted to an alternative PPI or esomeprazole 40 mg twice daily. EGD was repeated every six weeks to three months until the healing of the AU was diagnosed. If the patients with refractory AU were not able to stop smoking, at least a reduction of daily smoked cigarettes was intended. Revisional surgery was only considered when the AU was refractory for more than one year or more than two recurrences of AU appeared. Still, the indication for revisional surgery was a matter of patients' desire and was; therefore, not standardized.

## Results

There was no statistical difference in demographic data, preoperative findings in EGD or comorbidities between the CS and LS group. The results comparing the CS and LS group are depicted in Table 1.

**Table 1:** Demographic data, risk factors, comorbidities, type of gastric bypass surgery, incidence and treatment of anastomotic ulcers and follow-up in patients with circular-stapled anastomosis and in patients with linear-stapled anastomosis

	Circular-stapled anastomosis (= 126 patients)	Linear-stapled anastomosis (= 115 patients)	*p*
Demographic data			
Age (years)	38.9 ± 11.4	45.3 ± 12.6	0.12
Female	83 (65.9%)	81 (70.4%)	0.45
Male	43 (34.1%)	34 (29.6%)	0.45
BMI (kg/m^2^)	43.0 ± 6.8	42.7 ± 6.1	0.36
Risk factors			
Regular NSAID intake	0 (0%)	0 (0%)	N/A
Smoking	58 (44.4%)	44 (38.2%)	0.23
Comorbidities			
High blood pressure	40 (31.7%)	47 (40.9%)	0.14
Diabetes Type 2	20 (15.9%)	18 (15.7%)	0.96
H. pylori infection	22 (17.5%)	17 (14.8%)	0.57
GERD	29 (23%)	41 (35.7%)	0.1
Surgery			
Primary GBP	96 (76.2%)	72 (62.6%)	**0.011**
RYGB	96 (76.2%)	42 (36.5%)	**0.000**
OAGB*	-	30 (26.1%)	N/A
Revisional GBP	30 (23.8%)	43 (37.4%)	0.011
RYGB	30 (23.8%)	22 (19.1%)	0.81
OAGB*	-	21 (18.2%)	N/A
Suture at anastomosis	6 (4.8%)	4 (3.8%)	0.69
Anastomotic ulcer	**33 (26.2%)**	**13 (11.3%)**	**0.0034**
Incidence in Primary GBP	25 (26.0%)	9 (12.5%)	**0.011**
RYGB	25 (26.0%)	6 (14.2%)	**0.047**
OAGB	-	3 (10%)	N/A
Incidence in Revisional GBP	8 (26.6%)	4 (9.3%)	**0.03**
RYGB	8 (26.6%)	2 (9.1%)	**0.04**
OAGB	-	2 (9.5%)	N/A
Diagnosis (months after surgery)	11.2 ± 13.1	9.3 ± 7.1	0.31
Perforation	2 (1.6%)	2 (1.7%)	0.46
Conservative treatment	26 (20.6%)	11 (9.6%)	**0.017**
Planned redo	5 (4%)	0 (0%)	**0.011**
Follow-up			
Postoperative EGD	58 (46%)	36 (31.3%)	**0.009**
Time of last EGD (months after surgery)	23.4 ± 17.6	18.5 ± 12.1	0.053

OAGB was introduced in 2016 and only performed in linear-stapled anastomosis. *N/A* Not available, *BMI* body mass index, *NSAID* Nonsteroidal anti-inflammatory drug, *GERD* gastroesophageal reflux disease, *GBP* gastric bypass procedure, *RYGB* Roux-en-Y-gastric bypass, *OAGB* one-anastomosis gastric bypass, *EGD* oesophagogastroduodenoscopy

Altogether, 241 patients were included (females 68%, males 32%), 52.3% received a CS anastomosis and 47.7% LS anastomosis. In the CS group, 76.2% of the patients received a primary RYGB and 23.8% a revisional RYGB. There was no OAGB surgery in the CS group. In the LS group, 62.6% received a primary GBP (36.5% RYGB and 26.1% OAGB) and 37.4% received a revisional GBP (19.1% RYGB and 18.2% OAGB). All surgeries were performed laparoscopically and no major intraoperative complications occurred. Two patients required reoperation on postoperative day one: One patient in the CS group revealed an insufficiency of the jejuno-jejunostomy. One patient in the LS group had a perforation of the gastric remnant. There was no case of insufficiency of the gastrojejunostomy in both groups.

Altogether an AU occurred in 46 patients (19.1%). The incidence of AU was significantly higher in the CS group than in the LS group (26.2% in the CS group and 11.3% in the LS group, (*p* = 0.0034)). Of the 33 patients with AU in the CS group, 25 had undergone a primary RYGB and eight a revisional RYGB. Of the 13 patients in the LS group with AU, nine had received a primary GBP (six RYGB and three OAGB) and four a revisional GBP (two RYGB and two OAGB). In primary GBP, the incidence of AU was significantly higher in the CS group (26.0% vs 12.5%, *p* = 0.01), likewise in revisional surgeries only (26.6% vs 9.3%, *p* = 0.03). Moreover, patients undergoing revisional RYGB had a not significantly lower incidence of AU than primary ones (19.2% vs 22.4%, *p* = 0.31). The incidence of AU was lower in OAGB than in RYGB for primary surgeries (10% vs 22.4%, *p* = 0.03) and (not significantly) for revisional surgeries (9.5% vs19.2%, *p* = 0.18), respectively.

AU was diagnosed at a mean of 11.2 ± 13.1 months in the CS group and 9.3 ± 7.1 months in the LS group, respectively. In the CS group, three patients (2.4%) presented with perforated anastomotic ulcer and; therefore, required emergency surgery. Four patients (3.8%) had a redo of the gastrojejunostomy as a planned surgery due to refractory or recurrent AU after conservative treatment failure. One patient in the LS group (0.9%) required emergency surgery due to a perforated AU. There was one patient (0.9%) scheduled for a redo of the anastomosis due to a refractory AU in the LS group. The 37 remaining patients with a diagnosed AU (80.4%) were successfully treated conservatively.

Moreover, AU occurred significantly more often in patients who smoked than in non-smokers (69.6% and 35.9%; p = 0.0001). The amount of daily smoked cigarettes had no impact on the development of AU (mean 15.9 ± 7.6 daily smoked cigarettes in the AU group vs 16.9 ± 23.9 daily smoked cigarettes in the non-AU group). All patients with perforation, refractory or recurrent AU were active smokers (2–20 cigarettes daily).

There was no significant difference in gender, age, BMI, preoperative findings of EGD, medical comorbidities and medication between the AU and the non-AU group. All results comparing the AU group with the non-AU group are depicted in Table 2.

**Table 2:** Patients with anastomotic ulcer as compared to patients with no anastomotic ulcer

	No Anastomotic Ulcer (= 195 patients)	Anastomotic Ulcer (= 46 patients)	*p*
**Demographic data**			
** Age (years)**	41.1 ± 12.0	41.4 ± 13.8	0.42
** Female**	134 (68.7%)	30 (70.4%)	0.35
** Male**	61 (31.3%)	16 (29.6%)	0.35
BMI (kg/m^2^**)**	42.9 ± 6.4	42.6 ± 6.8	0.4
**Risk factors**			
** Regular NSAID intake**	0 (0%)	0 (0%)	N/A
** Smoking**	70 (35.9%)	32 (69.6%)	**0.0001**
** PPI intake**	62 (31.8%)	13 (28.3%)	0.36
**Comorbidities**			
** High blood pressure**	69 (35.4%)	18 (39.1%)	0.33
** Diabetes Type 2**	31 (15.9%)	7 (15.2%)	0.1
** H. pylori infection**	34 (17.4%)	5 (10.9%)	0.28
** GERD**	59 (30.1%)	11 (23.9%)	0.39
**Anastomosis**			
Circular-stapled ** Linear-stapled**	93 (47.7%) 102 (52.3%)	33 (71.7%) 13 (28.3%)	**0.0034** **0.0034**
**Surgery** ** Primary GBP**	134 (68.7%)	34 (73.9%)	0.28
** RYGB**	107 (79.9%)	31 (91.2%)	**0.029**
** OAGB**	27 (20.1%)	3 (8.8%)	**0.029**
** Revisional GBP**	61 (31.2%)	12 (26.1%)	0.24
** RYGB**	42 (68.9%)	10 (83.3%)	0.12
** OAGB**	19 (31.1%)	2 (16.7%)	0.12
** Suture at anastomosis**	10 (5.1%)	2 (4.3%)	0.41
**Follow-up**			
** Postoperative EGD**	50 (46%)	42 (100%)	**0.0001**
** Time of last EGD (months after surgery)**	18.3 ± 15.2	25.7 ± 15.8	**0.037**

Comparison of demographic data, risk factors, comorbidities, type of surgery and follow-up time. *N/A* not available, *BMI* body mass index, *NSAID* nonsteroidal anti-inflammatory drug, *GERD* gastroesophageal reflux disease, *GBP* gastric bypass procedure, *RYGB* Roux-en-Y-gastric bypass, *OAGB* one-anastomosis gastric bypass, *EGD* oesophagogastroduodenoscopy

## Discussion

The purpose of this study was to investigate whether a circular-stapled (CS) or a longitudinal-stapled (LS) gastrojejunostomy is different for the risk of development of anastomotic ulcers (AU). We could demonstrate that anastomotic ulcers appear significantly more often in a CS gastrojejunostomy than in an LS gastrojejunostomy.

Our study's strength was the standardized preoperative workup in a single tertiary centre, the continuity of the operation team and our study groups' demographic balance. Full information about our patients was provided because of consistent data documentation, the interdisciplinary arrangement of our treatment team and a low inter-hospital migration rate of Austrian patients. Moreover, follow-up examinations and EGD were performed by the same team over the observation period.

Our study's principal limit is the retrospective study design, which can only assume correlations for an AU development and can hardly identify any causality. Moreover, the detection of an AU during postoperative follow-up EGD was only possible in patients with symptoms of epigastric pain, reflux, melena or dyspepsia. Subclinical anastomotic ulcers may remain undetected. The follow-up time refers to our observation time between 01/2014 and 12/2018 and is therefore two to six years. Most AU occur within the first two years, but we know that they might occur for the first time even after more than ten years [27].

In our cohort, the overall incidence of AU was 19.1%. In the CS group, it was 26.2% and 11.3% in the LS group. Different studies report on an incidence of 0.6%–16%, including a prospective consecutive endoscopic observation study [4], a prospective multicentre study [28] and a recent cohort study from the Scandinavian obesity surgery registry (SOReg) [12]. Possible explanations for their relatively low number of AU (2,9% in the circular anastomosis group and 1% in the longitudinal anastomosis group) in their registry may be the primary use (97%) of a standardized linear-stapled anastomosis, a limitation in EGD follow-up and a reporting bias, as the authors note. Nevertheless, the odds ratio (OR) for developing an AU in the SOReg for CS is 3.10 (95%CI1.83–5.27) and therefore recommends a longitudinal anastomosis as well [11, 12]. The reason for ten times higher incidence of AU in our study as compared to SOReg remains unclear. The relatively high number of active smokers in our cohort, the more extended observation period and the easy access to EGD might explain the findings. Moreover, definitions for gastric ulcers cannot be transferred to anastomotic ulcers. Therefore, a redness or fibrin next to the stapler line is seen as AU in our study's symptomatic patients, but may not so in other studies.

Although the concept of weight loss surgery is well known since the pioneer times in 1960, operation methods and concepts changed over the year [29]. Up to date, there is no general recommended technique for pouch creation and limb length. From a functional aspect, the circular-stapled anastomosis was dominated by the idea of a restrictive component. A short and wide pouch was created, followed by a tight, circular-stapled anastomosis of 25 mm. The relatively tight anastomosis in the CS group leads to increased resistance and slower passage of the chyme from the pouch into the small bowel, which was supposed to restrict the food intake. However, restriction and malabsorption seem not to be the central functional aspect of weight loss in metabolic surgery [30]. With Rutledge's mini-gastric bypass introduction in 2001 [31] and the reported effects in weight loss and T2D remission rate, an elongated pouch became suitable. When we changed the CS into LS anastomosis, the shape of the anastomosis changed together with the pouch's size, the limb’s configuration and length.

Nevertheless, in the LS group, the restriction is maintained by the flow resistance of a long and narrow pouch according to the equation of Hagen-Poisseuille [32]. In addition, it has been advocated that a longer biliopancreatic limb has a better metabolic outcome [33, 34]. To optimize the metabolic effect, the biliary limb was extended from 100 to 150 cm and the alimentary limb was shortened to avoid malnutrition and keep a sufficient common channel length.

**Fig. 1:**
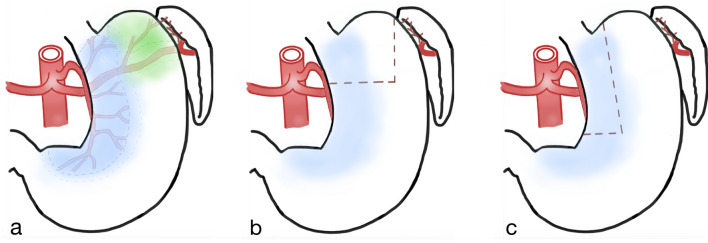
**a** Gastric area supplied by the left gastric artery (blue) and splenic artery (green). **b** Pouch of the circular anastomosis gastric bypass group (CS). **c** Pouch of the linear anastomosis gastric bypass group (LS). The blood supply from the splenic artery is regularly cut by the vertical staple line when the pouch is created.

A possible explanation for the higher incidence of AU in circular-stapled anastomosis might be the difference in the gastric pouch's shape and blood supply. In CS anastomosis, the pouch is short and wide, whereas in LS anastomosis, it is narrow and long. Branches of the left gastric artery mainly supply the upper part of the stomach on the right. The fundus is supplied primarily by branches of the splenic artery, the vasa gastricae breves (Fig. 1) [35]. When creating the pouch, blood flow from the left is regularly cut by the vertical staple line. Therefore, in a narrow pouch, there is enough blood supply from the left gastric artery and its aboral branches. The wider the pouch (such as in our CS series), the less blood supply is on the left side. Thus, reduced perfusion on the left side of a wide pouch may promote anastomotic ulceration.

Moreover, gastric acid secretion seems to be a leading causal agent for AU development, as successful treatment with antacids of the AU is frequently observed [3]. To this point, the correlation between the configuration of the pouch and the amount of gastric acid secretion is not clear. However, in a narrow pouch with less fundus, oxyntic cells are decreased and therefore less gastric acid secretion is conceivable [36].

Interestingly, H. pylori infection, GERD and gastric ulcers in the preoperative EGD did not impact the outcome of AU incidence in our study. All preoperatively detected upper-GI pathologies, especially erosive reflux oesophagitis, were treated with a PPI. Every single patient tested positive for biopsy-proven H. pylori was eradicated preoperatively. After eradication, a negative urea breath test or negative biopsies in EGD were requested before surgery. As we did not find a higher incidence of AU in successfully eradicated patients, we conclude that EGD with biopsies for H. pylori before surgery should be performed in all patients.

Concerning GERD, GBP is an excellent anti-reflux procedure in obese patients [37]. The increased abdominal pressure, a known risk factor for GERD, is reduced after losing weight. PPI was given to all our patients postoperatively for four weeks in a therapeutic dosage and was reduced to a prophylactic dosage for another eight weeks. In contrast to the literature, we did not observe a higher incidence of AU in patients with preoperative GERD [38, 39].

Above all, we found that smoking is a highly significant risk factor for the development of AU. Smoking is known to reduce micro-perfusion, which may lead to local ischaemia and damage. The number of daily smoked cigarettes is not crucial for the risk of developing AU, but smoking itself is [27]. A reduction of smoking is probably not enough. All our patients with perforation, refractory or recurrent AU scheduled for a redo operation were active smokers, indicating that smoking cessation may be the only chance for successful conservative treatment of the AU. Still, numerous (*n* = 70) patients did smoke, but did not develop an AU. In contrast, around 30% of the patients who developed an AU never smoked.

There was no higher incidence for developing an AU in patients with Type II diabetes (T2D) or arterial hypertension (HTN), assuming that adequately treated comorbidities do not impair the tissue's micro-perfusion.

According to the literature, NSAID intake is a risk factor for the development of AU [8]. Therefore, we educated our patients to renounce NSAID and take alternative pain killers. None of our patients with AU reported regular NSAID intake.

Literature has also shown that anastomotic strictures are higher in circular-stapled procedures [40–42]. Our study had two patients with anastomotic stricture (one in each group CS vs LS). To evaluate anastomotic strictures, a higher number of patients than in this study would be necessary. Still, it is to assume that persistent AU leads to strictures due to chronic inflammation. The low number of strictures in our study may reflect our strict management of AU diagnostic and therapy.

To sum up, the pathogenesis for the development of AU seems to be multifactorial. In our study comparing CS with wide pouch and LS with the narrow, longer pouch, AU was significantly more often found in CS. Consequently, the vascular supply seems to be the primary mechanism. On the one hand, a wide pouch has less arterial supply on the left (stapled) side. On the other hand, smoking, which causes reduced blood flow locally, is a significant risk factor for AU.

In our cohort, the introduction of LS came along with a change of the pouch configuration. From this aspect, it cannot be utterly differentiated if the better outcome is mainly based on the stapler used or the evolution of the pouch design. Further studies are needed to work up this question.

Finally, we believe that a linear-stapled procedure with a long and narrow pouch is the preferable procedure to avoid AU. In addition, the linear-stapled technique might minimize the risk of contamination and might reduce the operation time. Smoking cessation seems necessary to mitigate the risk for AU.

## Conclusion

Linear-stapled gastrojejunostomy with a long and narrow pouch is the preferred technique to reduce anastomotic ulcers. Moreover, smoking cessation before surgery is highly recommended.

## References

[1] Saarinen T, Meriläinen S, Koivukangas V (2019). Prospective randomized controlled trial comparing the efficacy and safety of Roux-en-Y gastric bypass and one-anastomosis gastric bypass (the RYSA trial): trial protocol and interim analysis. Trials.

[2] Wang F-G, Yan W-M, Yan M (2018). Outcomes of Mini vs Roux-en-Y gastric bypass: A meta-analysis and systematic review. Int J Surg.

[3] Steinemann DC, Bueter M, Schiesser M (2014). Management of anastomotic ulcers after Roux-en-Y gastric bypass: results of an international survey. Obes Surg.

[4] Csendes A, Torres J, Burgos AM (2011). Late marginal ulcers after gastric bypass for morbid obesity. Clinical and endoscopic findings and response to treatment. Obes Surg.

[5] Pyke O, Yang J, Cohn T (2019). Marginal ulcer continues to be a major source of morbidity over time following gastric bypass. Surg Endosc.

[6] Coblijn UK, Goucham AB, Lagarde SM (2014). Development of ulcer disease after Roux-en-Y gastric bypass, incidence, risk factors, and patient presentation: a systematic review. Obes Surg.

[7] Palmero M, Acquafresca PA, Rogula T (2015). Late surgical complications after gastric by-pass: a literature review. Arq Bras Cir Dig.

[8] Sverdén E, Mattsson F, Sondén A (2016). Risk Factors for Marginal Ulcer After Gastric Bypass Surgery for Obesity: A Population-based Cohort Study. Ann Surg.

[9] Rasmussen JJ, Fuller W, Ali MR (2007). Marginal ulceration after laparoscopic gastric bypass: an analysis of predisposing factors in 260 patients. Surg Endosc.

[10] Shope TR, Cooney RN, McLeod J (2003). Early results after laparoscopic gastric bypass: EEA vs GIA stapled gastrojejunal anastomosis. Obes Surg.

[11] Edholm D (2019). Systematic Review and Meta-analysis of Circular- and Linear-Stapled Gastro-jejunostomy in Laparoscopic Roux-en-Y Gastric Bypass. Obes Surg.

[12] Edholm D, Sundbom M (2015). Comparison between circular- and linear-stapled gastrojejunostomy in laparoscopic Roux-en-Y gastric bypass–a cohort from the Scandinavian Obesity Registry. Surg Obes Relat Dis.

[13] Edholm D, Ottosson J, Sundbom M (2016). Importance of pouch size in laparoscopic Roux-en-Y gastric bypass: a cohort study of 14,168 patients. Surg Endosc.

[14] El-Hayek K, Timratana P, Shimizu H (2012). Marginal ulcer after Roux-en-Y gastric bypass: what have we really learned?. Surg Endosc.

[15] Carr WRJ, Mahawar KK, Balupuri S (2014). An evidence-based algorithm for the management of marginal ulcers following Roux-en-Y gastric bypass. Obes Surg.

[16] Giordano S, Salminen P, Biancari F (2011). Linear stapler technique may be safer than circular in gastrojejunal anastomosis for laparoscopic Roux-en-Y gastric bypass: a meta-analysis of comparative studies. Obes Surg.

[17] Penna M, Markar SR, Venkat-Raman V (2012). Linear-stapled versus circular-stapled laparoscopic gastrojejunal anastomosis in morbid obesity: meta-analysis. Surg Laparosc Endosc Percutan Tech.

[18] Felsenreich DM, Bichler C, Langer FB, Gachabayov M, Eichelter J, Gensthaler L (2020). Surgical technique for one-anastomosis gastric bypass. Surg Technol Int..

[19] Schäfer A, Gehwolf P, Umlauft J (2019). Revisional gastric bypass after failed adjustable gastric banding-one-stage or two-stage procedure?. Obes Surg.

[20] Runkel M, Runkel N (2019). Esophago-gastric cancer after one anastomosis gastric bypass (OAGB). Chirurgia (Bucur).

[21] Saarinen T, Räsänen J, Salo J (2017). Bile reflux scintigraphy after mini-gastric bypass. Obes Surg.

[22] Guirat A, Addossari HM (2018). One anastomosis gastric bypass and risk of cancer. Obes Surg.

[23] Di Lorenzo N, Antoniou SA, Batterham RL (2020). Clinical practice guidelines of the European Association for Endoscopic Surgery (EAES) on bariatric surgery: update 2020 endorsed by IFSO-EC. EASO and ESPCOP Surg Endosc.

[24] Antoniou SA, Anastasiadou A, Antoniou GA (2017). Preoperative nutritional counseling versus standard care prior to bariatric surgery. Eur Surg.

[25] Gehwolf P, Hinder RA, DeVault KR (2015). Significant pressure differences between solid-state and water-perfused systems in lower esophageal sphincter measurement. Surg Endosc.

[26] Feng JJ, Gagner M, Pomp A (2003). Effect of standard vs extended Roux limb length on weight loss outcomes after laparoscopic Roux-en-Y gastric bypass. Surg Endosc.

[27] Azagury DE, Abu Dayyeh BK, Greenwalt IT (2011). Marginal ulceration after Roux-en-Y gastric bypass surgery: characteristics, risk factors, treatment, and outcomes. Endoscopy.

[28] Garrido AB, Rossi M, Lima SE (2010). Early marginal ulcer following Roux-en-Y gastric bypass under proton pump inhibitor treatment: prospective multicentric study. Arq Gastroenterol.

[29] Buchwald H, Buchwald JN (2002). Evolution of operative procedures for the management of morbid obesity 1950–2000. Obes Surg.

[30] Lutz TA, Bueter M (2014). The physiology underlying Roux-en-Y gastric bypass: a status report. Am J Physiol Regul Integr Comp Physiol.

[31] Rutledge R (2001). The mini-gastric bypass: experience with the first 1,274 cases. Obes Surg.

[32] Mortensen NA, Bruus H (2006). Universal dynamics in the onset of a Hagen-Poiseuille flow. Phys Rev E Stat Nonlin Soft Matter Phys.

[33] Murad AJ, Cohen RV, de Godoy EP (2018). A prospective single-arm trial of modified long biliopancreatic and short alimentary limbs Roux-En-Y gastric bypass in Type 2 diabetes patients with mild obesity. Obes Surg.

[34] Nergaard BJ, Leifsson BG, Hedenbro J (2014). e Gastric bypass with long alimentary limb or long pancreato-biliary limb–long-term results on weight loss, resolution of co-morbidities and metabolic parameters. Obes Surg.

[35] Loeweneck H, Feifel G (2014) Bauch. In: Lanz, Wachsmuth (ed) Praktische Anatomie (2/6), Band 2. Springer. ISBN-10: 3662580896

[36] Engevik AC, Kaji I, Goldenring JR (2020). The physiology of the gastric parietal cell. Physiol Rev.

[37] Gu L, Chen B, Du N (2019). Relationship between bariatric surgery and gastroesophageal reflux disease: a systematic review and meta-analysis. Obes Surg.

[38] Gilmore MM, Kallies KJ, Mathiason MA (2013). Varying marginal ulcer rates in patients undergoing laparoscopic Roux-en-Y gastric bypass for morbid obesity versus gastroesophageal reflux disease: Is the acid pocket to blame?. Surg Obes Relat Dis.

[39] Armstrong D (1999). Endoscopic evaluation of gastro-esophageal reflux disease. Yale J Biol Med.

[40] Takata MC, Ciovica R, Cello JP (2007). Predictors, treatment, and outcomes of gastrojejunostomy stricture after gastric bypass for morbid obesity. Obes Surg.

[41] Baccaro LM, Vunnamadala K, Sakharpe A (2015). Stricture rate after laparoscopic Roux-en-Y Gastric bypass with a 21-mm circular stapler versus a 25-mm linear stapler. Bariatr Surg Pract Patient Care.

[42] Lee S, Davies AR, Bahal S (2014). Comparison of gastrojejunal anastomosis techniques in laparoscopic Roux-en-Y gastric bypass: gastrojejunal stricture rate and effect on subsequent weight loss. Obes Surg.

